# 

**Published:** 1864-02

**Authors:** 


### CHICAGO MEDICAL JOURNAL.

**Terms, $2.00 per Annum.**

### CONTENTS OF THE FEBRUARY NUMBER.

PAGE.

ORIGINAL COMMUNICATIONS.

Are Varicose Veins a Cause of Post Partum Hœmorrhage? By J. W. Brooks, M. D., Chicago .......... 49

Clinical Lectures on Diseases of the Eye. By E. L. Holmes, M. D., of Chicago, Lecturer on Diseases of the Eye and Ear in Rush Medical College, etc.—The Ophthalmoscope .......... 55

Inversion of the Uterus. By Morton M. Eaton, M. D., of Peoria, Ill... 60

Correspondence .......... 62

Foreign Intelligence .......... 63

SELECTED.

On the Presence of Air in the Veins as a Cause of Death. B[illegible] Jas. Sumner Greene, M. D., of Dorchester, Mass .......... 65

Editorial and Miscellaneous.—The Sanitary Commission—The Surgeon General—Pharmacy in South America—Physiological Action of Baryta and Oxalic Acid—Ligature of the Subclavian—Inversion of the Uterus—Commencement in Rush Medical College.......... 82 to 91

Book Notices and Reviews .......... 92

Obituary Notices .......... 96

